# Psychopathological Profile Associated with Food Addiction Symptoms in Adolescents with Eating Disorders

**DOI:** 10.3390/ijerph20043014

**Published:** 2023-02-09

**Authors:** Michela Criscuolo, Giulia Cinelli, Ileana Croci, Ilenia Chianello, Anna Maria Caramadre, Alberto Eugenio Tozzi, Valeria Zanna

**Affiliations:** 1 Anorexia Nervosa and Eating Disorder Unit, Child Neuropsychiatry, Department of Neuroscience, Bambino Gesù Children’s Hospital IRCCS, 00165 Rome, Italy; 2 Predictive and Preventive Medicine Research Unit, Bambino Gesù Children’s Hospital IRCCS, 00165 Rome, Italy

**Keywords:** food addiction, YFAS 2.0, symptom count, eating disorders, anorexia nervosa, bulimia nervosa, adolescence

## Abstract

Eating disorders are considered one of the psychiatric disorders with a higher risk of death. Food addiction, related to some food addictive-like behaviours, is often in comorbidity with eating disorders and is associated with worse psychopathology. The present study aims to outline the food addiction profile, investigated using the Yale Food Addiction Scale 2.0 (YFAS 2.0), in 122 adolescents (median age: 15.6 years) suffering from eating disorders and to investigate its association with psychopathology. Patients filled out the Youth Self Report, the Multidimensional Anxiety Scale for Children 2, The Children Depression Inventory 2, and the Eating Disorder Inventory 3 (EDI-3). Pearson’s chi-square test and multiple correspondence analysis were used to identify profiles. The mean symptom count was 2.8 ± 2.7. The “withdrawal” symptom was the most frequent (51%) and the most associated with clinical scores. The diagnosis of bulimia nervosa and the EDI-3 bulimia scale resulted to be the only variables to be associated with positive YFAS 2.0 symptoms. Conversely, anorexia nervosa, restrictive and atypical, was not associated with YFAS 2.0 symptoms. In conclusion, outlining the food addiction profile of eating disorders may give information about a patient’s phenotype and could help to identify specific treatment models.

## 1. Introduction

Eating disorders (EDs) are considered one of the psychiatric disorders with a higher risk of death, with a 5 times higher mortality risk for patients diagnosed with anorexia nervosa (AN) compared to the general population [1]. It was estimated that in 2017, over 3.3 million healthy life years were lost to ED-related disability, and comorbidity may increase the burden of the disease worldwide [1].

EDs are often in comorbidity with both substance-related and addictive disorders (SRADs) [2] and food addiction (FA) [3,4,5,6,7]. It is an important issue, especially during adolescence, which is a period characterised by great developmental changes and a natural course of “instability”, with a risk of disordered and/or unhealthy eating behaviours and initiation of substance use [8,9]. In particular, the development of one disorder may constitute a vulnerability factor for the other, which may lead to the possibility of symptom switching [10]. The presence of untreated comorbid conditions is associated with a worse psychopathological picture [5] and complicates the evaluation, treatment, and prognosis of EDs [11,12,13].

The construct of FA has been recently introduced to explain some addictive-like behaviours characterised by excessive and dysregulated consumption of high-energy food. FA shares similar features with SRADs, which may reflect common underlying neural mechanisms and interacting substrates at neurobiological and metabolic levels [14,15,16]. At the same time, a debate about considering FA as an actual addictive disorder is still ongoing [17,18]. Nevertheless, FA is commonly defined according to the Diagnostic and Statistical Manual of Mental Disorders (DSM) [19] criteria for the SRADs and assessed by using the Yale Food Addiction Scale (YFAS), developed in 2009 [20] and revised in 2016 (YFAS 2.0) according to the DSM-5 [21]. The YFAS includes two scoring options: the symptom count, reflecting the number of fulfilled addiction-like symptoms, and the diagnosis for the presence of mild, moderate, or severe FA, which is identified when a threshold of three or more symptoms is met in addition to the impairment/distress criterion [21]. This last criterion reflects how food can affect a person’s daily life, which is mandatory to meet the diagnosis of FA. 

It is worth noting that it is possible to have a patient endorsing all the FA symptoms, but not meeting the mandatory criterion, and, therefore, not being classified as food addicted. It is likely that this kind of patient may be at risk and, if in comorbidity with an ED, may present a worse prognosis for recovery. For this reason, the use of the YFAS tool only to determine the absence/presence of FA appears reductive, considering that the number and type of symptoms endorsed by each patient could be informative and useful for treatment [22]. Nevertheless, many papers exclusively discuss FA diagnosis, while just a few studies report individual symptoms [22]. Considering clinical samples, recent reviews showed that the symptom count is higher, both in adolescents and adults, when compared to non-clinical ones [9,23]. Focusing on EDs, Albayrak et al. included this population in their study and reported the FA symptom count, but they used the old version of the YFAS based on DSM-5 [6].

In light of the literature evidence, and considering the importance of FA symptom endorsement in ED patients, this study aims to describe the symptom count and the frequency of FA symptoms among EDs subgroups in adolescence, using the latest version of the YFAS (YFAS 2.0). Moreover, it aims to outline ED patients’ FA profiles by investigating the association among FA symptoms, different ED diagnoses, and patient psychopathology. 

## 2. Materials and Methods

### 2.1. Patients and Study Design

This cross-sectional study analysed data from patients who were diagnosed with eating disorders and who were admitted to the Neuropsychiatry Unit at Bambino Gesù Children’s Hospital between March 2018 and May 2019. Male and female patients, aged 12 to 18 years old, with a primary diagnosis of an eating disorder based on DSM-5 criteria, were included in the study. Exclusion criteria were the presence of intellectual disabilities and a non-ED primary diagnosis. 

Upon hospital admission, all patients underwent a clinical evaluation, which is more fully described elsewhere [5]. 

This study was conducted in accordance with the Declaration of Helsinki. Patient consent was waived due to the difficulty in contacting all the patients who were no longer treated in the Hospital. The waiver was approved by the Ethics Committee of the Bambino Gesù Children’s Hospital (protocol code 1909_OPBG_2019, 30 September 2019). All the participants and their parents, when admitted to the Hospital, had signed a consent for the use of their clinical data for research purposes.

### 2.2. Psychopathological Measures

During the assessment procedure, all the patients completed self-administered psychopathological and eating questionnaires. For the analysis of the present study, the following questionnaires were used: the Youth Self-Report (YSR) [24,25], the Multidimensional Anxiety Scale for Children 2 (MASC 2) [26,27], the Children Depression Inventory 2 (CDI 2) [28,29], the Eating Disorder Inventory-3 (EDI-3) [30,31], and the Yale Food Addiction Scale 2.0 (YFAS-2.0) [21,32].

#### 2.2.1. Youth Self-Report 

The Italian version of the Youth Self-Report (YSR) questionnaire was administered to assess adolescents’ perceptions of their behaviour and emotional functioning [24]. This questionnaire, designed for 11- to 18-year-olds, consists of 112 items assessing behavioural, emotional, and social problems experienced by adolescents in the past 6 months. The YSR scores eight syndrome scales: Anxious/Depressed, Withdrawn/Depressed, Somatic Complaints, Social Problems, Thought Problems, Attention Problems, Aggressive Behaviour, and Rule-Breaking Behaviour. The Internalizing Problems scale is derived from the first three scales, while the Externalizing Problems scale is derived from the last two. The questionnaire also scores a Total Problems scale, which summarizes the results of the Internalizing and Externalizing scales. The Italian version of this assessment has demonstrated excellent day-to-day reliability, cross-informant agreement, and the ability to distinguish between referred and non-referred adolescents [25]. For the Internalizing, Externalizing, and Total Problems scales, values are considered clinical for a T score over or equal to 63. 

#### 2.2.2. Multidimensional Anxiety Scale for Children 2

The Multidimensional Anxiety Scale for Children 2 (MASC 2) [26] is a self-report test designed to assess anxiety in children and adolescents aged 8 to 19 years old. It consists of 50 items measuring Separation/Fears, Generalized Anxiety Index, Obsessions/Compulsions, Harm Avoidance, Social Anxiety (Humiliation/Rejection and Performance Fears), and Physical Symptoms (Panic and Tense/Restless). The Italian version of MASC 2 has demonstrated excellent validity, good internal consistency, and test–retest reliability [27]. Values are considered clinical for a T score over or equal to 70. 

#### 2.2.3. Children Depression Inventory 2

The Children Depression Inventory 2 (CDI 2) [28] is a self-report questionnaire used to assess depressive symptoms in children and adolescents aged 7 to 17 years old. It consists of 28 items, each of which includes three levels of symptom severity, ranging from 0 (absent) to 2 (defined, marked). The questionnaire scores two scales of Emotional and Functional Problems and a Total Score. It also provides scores on four sub-scales: Negative Mood/Physical Symptoms, Negative Self-Esteem, Ineffectiveness, and Interpersonal Problems. Statistical analysis has shown the good quality of the test items as well as their reliability and validity in the Italian version [29]. Values are considered clinical for a T score over or equal to 70. 

#### 2.2.4. Eating Disorder Inventory-3

The Eating Disorder Inventory-3 (EDI-3) [30] is a self-report questionnaire that measures psychological characteristics of clinical relevance in individuals who suffer from EDs. The instrument consists of 91 items. It scores three eating disorder-specific scales and nine general psychological scales, with different percentiles as cut-off scores for clinical scores (in parenthesis): Drive for Thinness—DT (>74°); Bulimia—B (>81°); Body Dissatisfaction—BD (>74°); Low Self-Esteem—LSE (>76°); Personal Alienation—PA (>72°); Interpersonal Insecurity—II (>74°); Interpersonal Alienation—IA (>74°); Interoceptive Deficits—ID (>71°); Emotional Dysregulation—ED (>73°); Perfectionism—P (>72°); Asceticism—A (>73°); Maturity Fears—MF (>64°). The Italian version of EDI-3 [31] has demonstrated excellent day-to-day reliability, cross-informant agreement, and good discriminating validity. 

#### 2.2.5. Yale Food Addiction Scale 2.0

The Yale Food Addiction Scale 2.0 (YFAS-2.0) is a self-reported test used to assess addiction-like eating behaviour [21,32]. A total of 35 questions are asked about the past 12 months with a score ranging from 0 (never) to 7 (every day). The test falls under a DSM-5 substance-related and addictive disorder symptom criterion or clinical impairment/distress and assesses 11 symptoms: (1) “amount”, using the substance in larger amounts or over a longer period than intended; (2) “attempts”, being unable to cut down or stop; (3) “time”, increased time and effort to obtain or use the substance or recover from its effects; (4) “activities”, reduction of social, occupational, or recreational activities because of substance use; (5) “consequences”, using the substance despite a persistent physical or psychological problem caused or exacerbated by the substance; (6) “tolerance”, consuming increasing amounts of a substance to achieve the same effects or experiencing diminished effects with continued use of the same amounts; (7) “withdrawal”, withdrawal symptoms when the substance is not consumed or using the substance to avoid withdrawal symptoms; (8) “problems”, continued substance use despite social or interpersonal problems caused or exacerbated by substance use; (9) “obligations”, failure to fulfil major role obligations at work, school, or home as a result of substance use; (10) “situations”, recurrent substance use in situations in which it is physically hazardous; (11) “craving”, a persistent desire or unsuccessful efforts to cut down substance use.

The instrument provides no sum score. The YFAS 2.0 symptom count is calculated as the sum of the number of fulfilled diagnostic criteria (ranging from 0 to 11). Therefore, for the “diagnosis” scoring option, both the symptom count score and the clinical significance criterion are used:

No Food Addiction = 1 or fewer symptoms/Does not meet criteria for impairment/distress criteria; Mild Food Addiction = 2 or 3 symptoms and impairment/distress criteria;

Moderate Food Addiction = 4 or 5 symptoms and impairment/distress criteria;

Severe Food Addiction = 6 or more symptoms and impairment/distress criteria.

### 2.3. Statistical Analysis

Categorical variables are presented as numbers and percentages in parentheses (%), and continuous variables are presented as the median and interquartile range in parentheses (IQR). Pearson’s chi-square test was used to evaluate the association between the presence of FA symptoms and clinical scores on the YSR, MASC 2, CDI 2, and EDI-3 scales. Statistical significance was set at *p* < 0.05. Multiple correspondence analysis (MCA) was performed to identify the variables associated with FA symptoms. The following variables were used to build the graph (“active” variables): ED diagnosis (ARFID, avoidant restrictive food intake disorder; R-AN, restrictive anorexia nervosa; A-AN, atypical anorexia nervosa; BP-AN, binge purging anorexia nervosa; BN, bulimia nervosa; BED, binge eating disorder; ED-NOS, eating disorder not otherwise specified), YSR composite scales (Internalizing, Externalizing and Total Problems), and total score for MASC 2, CDI 2, and EDI-3 primary scales. The scores of the psychopathological tests were used as dichotomous variables (clinical/non-clinical). As only one patient was diagnosed with BED and one with BP-AN, these subgroups were excluded from the MCA analysis due to an insufficient number of patients. 

Data analysis was carried out using STATA 17.0 (StataCorp, College Station, TX, USA).

## 3. Results

### 3.1. Patients

From the initial cohort of 122 patients aged 12–18 years diagnosed with EDs, 15 were excluded for a non-ED primary diagnosis, while 5 were excluded due to missing psychopathological data. Finally, 102 patients (94 females, 92.2%) were included in the analysis for the present study. The patients’ characteristics are shown in Table 1. 

### 3.2. Food Addiction Profile and ED Subgroups

Considering the whole sample, 53 patients (51.9%) met the criteria for the diagnosis of FA, of which 19 (18.6%), 15 (14.7%) and 19 (18.6%) were diagnosed with mild, moderate, and severe FA, respectively. The mean symptom count was 2.8 (standard deviation 2.7). Table 2 shows the YFAS 2.0 symptom median scores and the frequency over the ED subgroups. Patients with BED and BN showed the highest symptom count. 

For almost all the ED subgroups, except for ARFID and BP-AN (which count only one patient), the “withdrawal” symptom was the most frequent. For patients diagnosed with ARFID, “time” resulted to be the most frequent symptom, while the one patient diagnosed with BP-AN only endorsed the “time” and “problems” symptoms. 

### 3.3. Association between Food Addiction and Psychopathological Symptoms

As shown in Table 3, the presence of all the addiction symptoms, except for “time” and “problems”, correlated with clinical scores on the EDI-3 bulimia scale.

The “withdrawal” symptom was the one most associated with clinical scores on the psychopathological questionnaires. In addition, “problems” was the other symptom most correlated with clinical scores.

The “time” and “consequences” symptoms were the only ones associated with anxiety (MASC total score). 

Tables S1 and S2 show the complete analysis.

### 3.4. Multiple Correspondence Analysis (MCA)

**Figure 1:**
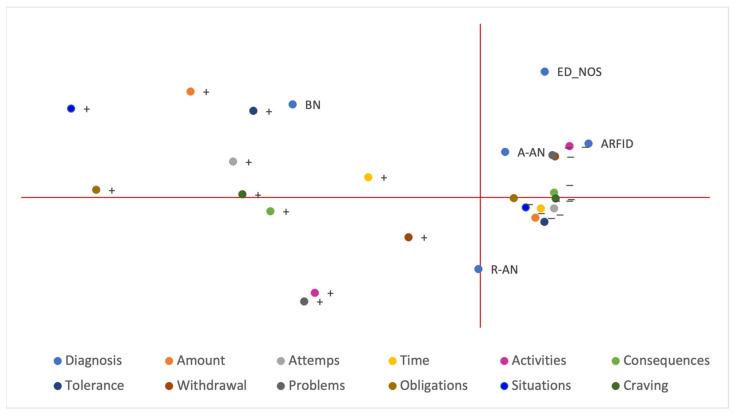
Multiple correspondence analysis between FA symptoms and ED diagnosis. As only one patient was diagnosed with binge eating disorder and one with binge purging-anorexia nervosa, these subgroups were excluded from the MCA analysis due to an insufficient number of patients. + and–correspond to positivity and negativity to FA symptoms, respectively. A-AN, atypical anorexia nervosa; ARFID, avoidant restrictive food intake disorder; BN, bulimia nervosa; ED-NOS, eating disorder not otherwise specified; EDs, eating disorders, R-AN, restrictive anorexia nervosa.

As shown in Figure 1, BN patients were associated with a profile characterised by positivity to the different FA symptoms, most strongly to “amount”, “attempts” and “tolerance”.

ARFID and A-AN diagnoses shared the same profile of negativity to FA symptoms, in particular to “activities”, “problems”, and “withdrawal”. In addition, the ED-NOS patients’ results were close to this type of profile. 

An R-AN diagnosis was not associated with specific FA symptoms. 

Regardless of the diagnosis, positivity to the “activities” and “problems” symptoms was strongly associated, and they tended to occur together. 

The percentage of explained variance was 87.7% for the model.

**Figure 2:**
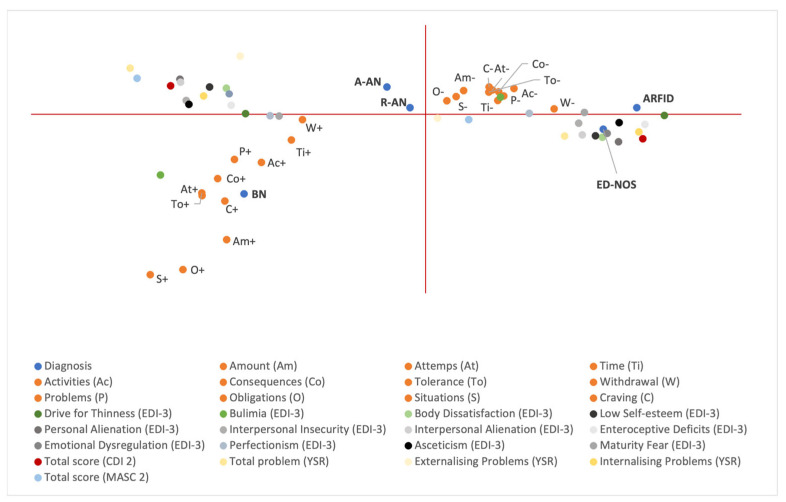
Multiple correspondence analysis using FA symptoms, ED diagnosis, and psychopathological scores. As only one patient was diagnosed with binge eating disorder and one with binge purging-anorexia nervosa, these subgroups were excluded from the MCA analysis due to an insufficient number of patients. + and - correspond to positivity and negativity to FA symptoms, respectively. A-AN, atypical anorexia nervosa; ARFID, avoidant restrictive food intake disorder; BN, bulimia nervosa; ED-NOS, eating disorder not otherwise specified; EDs, eating disorders, R-AN, restrictive anorexia nervosa.

Figure 2 shows the results of the MCA analysis after adding the score of the psychopathological questionnaires as “active” variables.

All the positive reported YFAS symptoms were grouped together (III quadrant), as were the negative ones (I quadrant). The only psychopathological scale associated with these symptoms is the bulimia scale from the EDI-3 test. A clinical score is associated with positivity to the symptoms and vice versa. In addition, BN remains the only diagnosis to be associated with the positive YFAS 2.0 symptoms, showing a strong association with the “craving” symptom.

Conversely, the ARFID diagnosis result was associated with negative YFAS symptoms. Finally, AN (both restrictive and atypical) was not associated with YFAS symptoms at all but was associated with all the clinical scores of the psychopathological tests (grouped in the II quadrant).

All the non-clinical psychopathological tests were grouped together in the IV quadrant and were associated with the ED-NOS diagnosis. 

The percentage of explained variance was 89.6% for the model.

## 4. Discussion

The first aim of the present study was to report the mean symptom scores and to describe the frequency of FA symptom endorsement among the ED subgroups. 

In the analysed sample, the mean symptom count was 2.8 (SD 2.7). These findings differ from a previous study by Albayrak et al. [6], which used the previous version of the YFAS. When considering only the patients with ED (*n* = 37), they found a mean symptom count of 2.97 (SD 1.83) over a total of seven symptoms instead of eleven. It is worth noting, however, that the old YFAS counted the impairment/distress criterion as one of the seven symptoms, while it is not included in the symptom count for YFAS 2.0. Since 73% of the patients included in the study by Albayrak et al. [6] endorsed this criterion, it likely had a weight in raising the mean value of the symptom count.

When considering a non-clinical sample, Aloi et al. [33] found a mean symptom count of 1.3 (SD 2.3) in adolescents and young adults (17–24 years) using YFAS 2.0. Even though that study did not distinguish different age groups, results from the present research may confirm a higher symptom count in the clinical samples compared to non-clinical ones [9]. 

A higher symptom count emerged in BN patients. This is in line with the literature that describes the highest prevalence rates of YFAS 2.0 diagnoses in BN patients [34]. 

In the present study, the most frequently endorsed YFAS 2.0 symptom was “withdrawal” (51%) followed by “times” and “activities”. Conversely, Albayrak et al. [6], in their subgroup of ED patients, found that only 35% endorsed the “withdrawal” symptom, while all endorsed the symptom ‘persistent desire or unsuccessful effort to cut down or control eating’, which corresponds to “attempts” in YFAS 2.0 and was endorsed only by 19% of patients in the present study. ‘Use despite negative physical or psychological problems’ (“consequences”) and ‘giving up important activities’ (“activities”) were the other two most frequent symptoms (43.2 and 37.8%, respectively) in the research by Albayrak et al. [6]. These symptoms were only endorsed by 26,5% and 34.4% of patients in the present study. In the non-clinical adolescent samples, the YFAS 2.0 symptoms of “amount” (13.7%), “attempts” (12.2%), and “problems” (11%) were shown to be the most endorsed [35]. 

In the sample of the present study, the “withdrawal” symptom was the most frequent, except for ARFID and BP-AN (which count only one patient). This symptom refers to the cascade of aversive physical, affective, and cognitive symptoms that occur when the use of an addictive substance is reduced or stopped [19]. It seems that children with high palatable FA may experience a withdrawal syndrome when their parents restrict access to these specific foods [36]. Similarly, it is possible that the restrictive eating pattern characteristic of the sample in the present study could have solicited feelings related to withdrawal, above all because restriction lasted over time. 

The second aim of this study was to outline ED patients’ FA profiles by investigating the association among YFAS 2.0 symptoms, the different ED diagnoses, and patients’ psychopathology. The results showed that the positivity to almost all YFAS 2.0 symptoms was associated with the clinical score on the EDI-3 bulimia scale. In addition, seven symptoms were associated with the drive for thinness scale. Both scales shared an association with the symptom “amount”, probably due to the sensation of eating an excessive quantity of food even in those patients with restrictive eating behaviour. Conversely, these two scales differed for the symptom “problems”, associated only with a drive for thinness, and the symptoms “obligations”, “situations” and “craving”, associated only with bulimia. These two EDI-3 scales are useful to discern the two main dysfunctional eating patterns, characterised by restriction or dyscontrol, so it is possible that using YFAS 2.0 symptoms could help distinguish the different phenotypes correlated to them and in guiding therapeutic decisions. 

The MCA analysis was performed to better investigate the association among each symptom of FA. The percentages of explained variance did not change after adding the psychopathological questionnaires to the analysis. Hence, the results showed that psychopathological scores do not have a statistical weight in FA profiling. Moreover, the findings showed a strong association between all FA symptoms and a BN diagnosis, especially for “craving”. This is in line with previous studies [22]. Conversely, the ARFID diagnosis was associated with negativity to all FA symptoms, while AN (both restrictive and atypical) was not associated with YFAS 2.0 symptoms at all but was associated with all the clinical scores of the psychopathological tests. Regarding the psychopathological tests, the EDI-3 bulimia scale was found to be the only one associated with positivity to the YFAS 2.0 symptoms.

These results potentially highlight two different phenotypes of FA, as suggested by Wiss et al. [37]. AN patients are characterised by withdrawal symptoms, due to their strict diet restriction, and may feel incapable of maintaining the restraint behaviour. This may result in a high score for the symptom count and a diagnosis of FA, especially in those cases in which the restriction persists for a prolonged period. Nevertheless, the MCA analysis shows that these patients could be considered “false positives”. Conversely, BN patients may represent the “true positives” for a FA diagnosis. 

Withdrawal, which emerges as the most frequent symptom, may be linked to the psychological condition deriving from food restriction and, therefore, may be a negative consequence, as with dysphoria or dysthymia [38]. Following the idea of “false positivity” to FA, it is possible that patients with AN were addicted not to the food itself, but to the sensation linked to the gratification following fasting (negative association), while patients with BN may have a real addiction related to food.

This distinction may be important in clinical practice. Wiss et al. suggest considering the two phenotypes for nutritional intervention: in the case of a “true” FA, it could be useful to sustain specific addictive food avoidance, while in the case of a “false” FA, it could be crucial to contrast the restrictive behaviour and patient to all food [37].

The fact that FA positivity was evidenced not only in patients with binge purging behaviour or overweight subjects but also in patients with restrictive eating disorders [4,5,39], supports the hypothesis that FA is a transdiagnostic construct [7,40,41,42]. Several studies have noticed that a significant percentage of patients with R-AN switch to BP-AN during the course of the pathology. Going deeper into this phenomenon, Sanchez et al. [43] found common elements in the clinical profiles of R-AN patients with FA positivity and BP-AN patients, suggesting that FA positivity may sustain a possible cross-over from one subtype to another. Moreover, a review by Skinner et al. [9] outlined the co-occurrence of FA with other mental health conditions in children and adolescents. So, having information about the presence of FA can inform clinicians about the patient’s overall mental health and their risk of transitioning to binge–purge behaviours and help to adapt the treatment to their clinical and personality characteristics.

The main limitations of the present study are the small sample size and, in particular, the difference between the ED subgroup numerosity with BP-AN and BED counting only one patient each. Therefore, the population considered may not be representative of universal adolescent EDs and the results may not be generalizable. In addition, the study lacks a control group of healthy adolescents. Furthermore, in the sample of the present study, there were only two patients who had previous comorbidity with substance abuse, and due to the poor numerosity, it was not possible to perform any analysis on them.

## 5. Conclusions

In conclusion, the results of this study, together with the above observations, suggest that outlining the FA addiction profile of a patient with an ED diagnosis may give information about the specific phenotype and the pathology’s trajectory and could help clinicians to identify the most suitable and tailored treatment care model.

This is the first study investigating the FA profile of ED patients considering the symptom count and the different symptom endorsements, instead of the diagnosis, using the latest version of YFAS 2.0. Further studies on FA, including a greater number of adolescent patients diagnosed with BED and BP-AN, are needed. Likewise, studies on EDs in comorbidity with SRADs could be useful to better understand the symptomatology shared between different addiction profiles. Finally, future research may go into further detail regarding the difference between R-AN and BP-AN in order to understand the conditions associated with FA positivity. In addition, for A-AN patients, the hypothesis about FA being derived from restricted behaviour should be investigated in association with the entity and rapidity of weight loss and BMI.

## Figures and Tables

**Table 1:** Patients’ characteristics.

Variable	*n* = 102
Age	15.6 (14.00–16.7)
Weight (kg)	43.6 (38.6–51.0)
Height (cm)	159.2 (154.5–164.0)
BMI	17.3 (15.7–19.1)
BMI percentile	10 (1.0–33.0)
Diagnosis	
ARFID	7 (6.9)
R-AN	51 (50.0)
A-AN	21 (20.6)
BP-AN	1 (1.0)
BN	10 (9.8)
BED	1 (1.0)
ED-NOS	11 (10.8)

Values are expressed as median and IQR (M (IQR)) for continuous variables or as number and percentage (*n* (%)) for categorical variables. A-AN, atypical anorexia nervosa; ARFID, avoidant restrictive food intake disorder; BED, binge eating disorder; BMI, body mass index; BN, bulimia nervosa; BP-AN, binge purging anorexia nervosa; ED-NOS, eating disorder not otherwise specified; EDs, eating disorders, R-AN, restrictive anorexia nervosa.

**Table 2:** Food addiction symptoms profile in the ED subgroups.

	Whole Sample (*n* = 102)	ARFID (*n* = 7)	R-AN (*n* = 51)	A-AN (*n* = 21)	BP-AN (*n* = 1)	BN (*n* = 10)	BED (*n* = 1)	ED-NOS (*n* = 11)
Symptom count	2 (1–4)	1 (0–4)	2 (1–4)	2 (1–4)	2 (−)	4.5 (1–8)	9 (-)	1 (0–2)
Amount	17 (16.7)	1 (14.3)	4 (7.8)	3 (14.3)	0 (0.0)	6 (60.0)	1 (100.0)	2 (18.2)
Attempts	24 (19.6)	0 (0.0)	13 (25.5)	5 (23.8)	0 (0.0)	3 (30.0)	1 (100.0)	2 (18.2)
Time	37 (36.3)	3 (42.7)	17 (33.3)	8 (38.1)	1 (100.0)	5 (50.0)	1 (100.0)	2 (18.2)
Activities	35 (34.4)	2 (28.6)	20 (39.2)	6 (28.6)	0 (0.0)	6 (60.0)	0 (0.0)	1 (9.1)
Consequences	27 (26.5)	1 (14.3)	15 (29.4)	3 (14.3)	0 (0.0)	5 (50.0)	1 (100.0)	2 (18.2)
Tolerance	23 (22.5)	1 (14.3)	10 (19.6)	5 (23.8)	0 (0.0)	4 (40.0)	1 (100.0)	2 (18.2)
Withdrawal	52 (51.0)	2 (28.6)	24 (47.1)	13 (61.9)	0 (0.0)	7 (70.0)	1 (100.0)	5 (45.4)
Problems	31 (30.4)	1 (14.3)	19 (37.2)	4 (19.0)	1 (100.0)	3 (30.0)	1 (100.0)	2 (18.2)
Obligations	8 (7.8)	0 (0.0)	3 (5.9)	3 (14.3)	0 (0.0)	1 (10.0)	0 (0.0)	1 (9.1)
Situations	11 (10.8)	0 (0.0)	5 (9.8)	2 (9.5)	0 (0.0)	2 (20.0)	1 (100.0)	1 (9.1)
Craving	25 (24.5)	1 (14.3)	14 (27.4)	2 (9.5)	0 (0.0)	5 (50.0)	1 (100.0)	2 (18.2)

Values are expressed as median and IQR (M (IQR)) for continuous variables or as number and percentage (*n* (%)) for categorical variables. A-AN, atypical anorexia nervosa; ARFID, avoidant restrictive food intake disorder; BED, binge eating disorder; BN, bulimia nervosa; BP-AN, binge purging anorexia nervosa; ED-NOS, eating disorder not otherwise specified; EDs, eating disorders, R-AN, restrictive anorexia nervosa.

**Table 3:** Association between FA symptoms and psychopathological scales.

	Amount	Attempts	Time	Activities	Consequences	Tolerance	Withdrawal	Problems	Obligations	Situations	Craving
Internalising Problems (YSR)			0.010	0.010	0.002		0.001	0.008			
Externalising Problems (YSR)											
Total Problems (YSR)			0.017				0.001	0.014			
Total score (MASC 2)			0.043		0.017						
Total score (CDI 2)				0.029	0.009		0.009	0.030			
Drive for Thinness (EDI-3)	0.020	0.040		0.001	0.035	0.019	0.010	<0.001			
Bulimia (EDI-3)	<0.001	0.010		0.011	0.008	<0.001	0.043		0.005	0.007	<0.001
Body dissatisfaction (EDI-3)											
Low Self-esteem (EDI-3)											
Personal Alienation (EDI-3)							0.044				
Interpersonal Insecurity (EDI-3)		0.001				0.011	0.037	0.023		0.013	
Interpersonal Alienation (EDI-3)							0.037				
Interoceptive Deficits (EDI-3)		0.013		0.022	0.001		0.003	0.048			
Emotional Dysregulation (EDI-3)							0.017	0.002			
Perfectionism (EDI-3)		0028				0.028					
Asceticism (EDI-3)				0.029		0.012	0.027	0.009			0.002
Maturity Fear (EDI-3)		0.026	0.034				0.010				0.001

The Pearson’s chi-square test was used to evaluate the association between the presence of FA symptoms and clinical scores on the YSR, EDI-3, MASC 2, and CDI 2 scales. Statistical significance for *p* < 0.05. CDI 2, Children Depression Inventory 2; EDI-3, Eating Disorder Inventory-3; MASC 2, Multidimensional Anxiety Scale for Children 2; YSR, Youth Self Report.

## Data Availability

Not applicable.

## Supplementary Materials

The following supporting information can be downloaded at: https://www.mdpi.com/article/10.3390/ijerph20043014/s1, Table S1: Correlation between FA symptoms and psychopathological scales (pt. 1); Table S2. Correlation between FA symptoms and psychopathological scales (pt. 2).
