# Supplementary material for: Psychopathological Profile Associated with Food Addiction Symptoms in Adolescents with Eating Disorders
Source: Int J Environ Res Public Health. 2023 Feb 9;20(4):3014. doi: 10.3390/ijerph20043014 (PMC9960227; doi:10.3390/ijerph20043014)
Supplement: Supplementary file 1 [file ijerph-20-03014-s001.zip › ijerph-2168103-supplementary.pdf]

International Journal of
Environmental Research and Public Health

MDPI

Table S1. Association between FA symptoms and psychopathological scales (pt.1)

	Amount			Attempts			Time			Activities			Consequences			Tolerance		
	-	+	p	-	+	p	-	+	p	-	+	p	-	+	p	-	+	p
**Internalising Problems (YSR)**	40 (47.1)	9 (52.9)	0.658	33 (42.9)	16 (64.0)	0.066	25 (38.5)	24 (64.9)	0.010	26 (38.8)	23 (65.7)	0.010	29 (38.7)	20 (74.1)	0.002	34 (43.0)	15 (65.2)	0.061
**Externalising Problems (YSR)**	5 (5.9)	1 (5.9)	1.000	3 (3.9)	3 (12.0)	0.135	3 (4.6)	3 (8.1)	0.471	3 (4.5)	3 (8.6)	0.404	4 (5.3)	2 (7.4)	0.694	6 (7.6)	0 (0.0)	0.173
**Total Problems (YSR)**	27 (31.8)	5 (29.4)	0.849	22 (28.6)	10 (40.0)	0.285	15 (23.1)	17 (45.9)	0.017	18 (26.9)	14 (40.0)	0.175	22 (29.3)	10 (37.0)	0.459	24 (30.4)	8 (34.8)	0.689
**Drive for Thinness (EDI-3)**	44 (51.8)	14 (82.4)	0.020	41 (53.2)	17 (68.0)	0.196	33 (50.8)	25 (67.6)	0.100	30 (44.8)	28 (80.0)	0.001	38 (50.7)	20 (74.1)	0.035	40 (50.6)	18 (78.3)	0.019
**Bulimia (EDI-3)**	12 (14.1)	11 (64.7)	<0.001	10 (13.0)	13 (52.0)	<0.001	11 (16.9)	12 (32.4)	0.072	10 (14.9)	13 (37.1)	0.011	12 (16.0)	11 (40.7)	0.008	11 (13.9)	12 (52.2)	<0.001
**Body dissatisfaction (EDI-3)**	39 (45.9)	9 (52.9)	0.595	38 (49.4)	10 (40.0)	0.416	26 (40.0)	22 (59.5)	0.058	28 (41.8)	20 (57.1)	0.140	34 (45.3)	14 (51.9)	0.561	35 (44.3)	13 (56.5)	0.302
**Low Self-esteem (EDI-3)**	37 (43.5)	8 (47.1)	0.789	33 (42.9)	12 (48.0)	0.653	27 (41.5)	18 (48.6)	0.487	28 (41.8)	17 (48.6)	0.513	29 (38.7)	16 (59.3)	0.065	31 (39.2)	14 (60.9)	0.066
**Personal Alienation (EDI-3)**	37 (43.5)	8 (47.1)	0.789	32 (41.6)	13 (52.0)	0.361	27 (41.5)	18 (48.6)	0.487	27 (40.3)	18 (51.4)	0.282	30 (40.0)	15 (55.6)	0.163	32 (40.5)	13 (56.5)	0.173
**Interpersonal Insecurity (EDI-3)**	31 (36.5)	8 (47.1)	0.412	27 (35.1)	12 (48.0)	0.248	23 (35.4)	16 (43.2)	0.432	21 (31.3)	18 (51.4)	0.048	25 (33.3)	14 (51.9)	0.090	25 (31.6)	14 (60.9)	0.011
**Interpersonal Alienation (EDI-3)**	32 (37.6)	7 (41.2)	0.785	26 (33.8)	13 (52.0)	0.103	22 (33.8)	17 (45.9)	0.227	22 (32.8)	17 (48.6)	0.121	26 (34.7)	13 (48.1)	0.216	28 (35.4)	11 (47.8)	0.282
**Interoceptive Deficits (EDI-3)**	42 (49.4)	12 (70.6)	0.110	38 (49.4)	16 (64.0)	0.202	30 (46.2)	24 (64.9)	0.069	30 (44.8)	24 (68.6)	0.022	32 (42.7)	22 (81.5)	0.001	38 (48.1)	16 (69.6)	0.070
**Emotional Dysregulation (EDI-3)**	40 (47.1)	9 (52.9)	0.658	37 (48.1)	12 (48.0)	0.996	28 (43.1)	21 (56.8)	0.184	29 (43.3)	20 (57.1)	0.183	33 (44.0)	16 (59.3)	0.174	35 (44.3)	14 (60.9)	0.162
**Perfectionism (EDI-3)**	31 (36.5)	9 (52.9)	0.204	26 (33.8)	14 (56.0)	0.048	23 (35.4)	17 (45.9)	0.294	23 (34.3)	17 (48.6)	0.162	27 (36.0)	13 (48.1)	0.268	27 (34.2)	13 (56.5)	0.053
**Asceticism (EDI-3)**	36 (42.4)	10 (58.8)	0.213	28 (36.4)	18 (72.0)	0.002	25 (38.5)	21 (56.8)	0.074	25 (37.3)	21 (60.0)	0.029	31 (41.3)	15 (55.6)	0.203	31 (39.2)	15 (65.2)	0.028
**Maturity Fears (EDI-3)**	43 (50.6)	9 (52.9)	0.859	32 (41.6)	20 (80.0)	0.001	28 (43.1)	24 (64.9)	0.034	28 (41.8)	24 (68.6)	0.100	34 (45.3)	18 (66.7)	0.057	35 (44.3)	17 (74.0)	0.012
**Total Problems (MASC 2)**	11 (12.9)	2 (11.8)	0.894	9 (11.7)	4 (16.0)	0.574	5 (7.7)	8 (21.6)	0.043	6 (9.0)	7 (20.0)	0.112	6 (8.0)	7 (25.9)	0.017	8 (10.1)	5 (21.7)	0.142
**Total score (CDI 2)**	37 (43.5)	9 (52.9)	0.477	31 (40.3)	15 (60.0)	0.085	25 (38.5)	21 (56.8)	0.074	25 (37.3)	21 (60.0)	0.029	28 (37.3)	18 (66.7)	0.009	32 (40.5)	14 (60.9)	0.084

The Pearson's chi-square test was used to evaluate the association between the presence of FA's symptoms (+ present; - absent) and clinical scores at YSR, EDI-3, MASC 2 and CDI 2 scales. Statistical significance for p<0-05. CDI 2, Children Depression Inventory 2; EDI-3, Eating Disorder Inventory- 3; MASC 2, Multidimensional Anxiety Scale for Children 2; YSR, Youth Self Report.

Table S2. Association between FA symptoms and psychopathological scales (pt.2)

	Withdrawal			Problems			Obligations			Situations			Craving		
	-	+	p	-	+	p	-	+	p	-	+	p	-	+	p
**Internalising Problems (YSR)**	16 (32.0)	33 (63.5)	0.001	28 (39.4)	21 (67.7)	0.008	45 (47.9)	4 (50.0)	0.908	43 (47.3)	6 (54.5)	0.647	33 (42.9)	16 (64.0)	0.066
**Externalising Problems (YSR)**	1 (2.0)	5 (9.6)	0.102	4 (5.6)	2 (6.5)	0.872	5 (5.3)	1 (12.5)	0.407	6 (6.6)	0 (0.0)	0.380	3 (3.9)	3 (12.0)	0.135
**Total Problems (YSR)**	8 (16.0)	24 (46.2)	0.001	17 (23.9)	15 (48.4)	0.014	29 (30.9)	3 (37.5)	0.697	28 (30.8)	4 (36.4)	0.706	22 (28.6)	10 (40.0)	0.285
**Drive for Thinness (EDI-3)**	22 (44.0)	36 (69.2)	0.010	31 (43.7)	27 (87.1)	<0.001	51 (54.3)	7 (87.5)	0.068	51 (56.0)	7 (63.6)	0.631	41 (53.2)	17 (68.0)	0.196
**Bulimia (EDI-3)**	7 (14.0)	16 (30.8)	0.043	13 (18.3)	10 (32.3)	0.121	18 (19.1)	5 (62.5)	0.005	17 (18.7)	6 (54.5)	0.007	10 (13.0)	13 (52.0)	<0.001
**Body dissatisfaction (EDI-3)**	19 (38.0)	29 (55.8)	0.072	30 (42.3)	18 (58.1)	0.141	43 (45.7)	5 (62.5)	0.362	43 (47.3)	5 (45.5)	0.910	38 (49.4)	10 (40.0)	0.416
**Low Self-esteem (EDI-3)**	18 (36.0)	27 (51.9)	0.105	29 (40.8)	16 (51.6)	0.314	39 (41.5)	6 (75.0)	0.067	41 (45.1)	4 (36.4)	0.583	33 (42.9)	12 (48.0)	0.653
**Personal Alienation (EDI-3)**	17 (34.0)	28 (53.8)	0.044	28 (39.4)	17 (54.8)	0.150	42 (44.7)	3 (37.5)	0.695	39 (42.9)	6 (54.5)	0.461	32 (41.6)	13 (52.0)	0.361
**Interpersonal Insecurity (EDI-3)**	14 (28.0)	25 (48.1)	0.037	22 (31.0)	17 (54.8)	0.023	36 (38.3)	3 (37.5)	0.964	31 (34.1)	8 (72.7)	0.013	27 (35.1)	12 (48.0)	0.248
**Interpersonal Alienation (EDI-3)**	14 (28.0)	25 (48.1)	0.037	25 (35.2)	14 (45.2)	0.342	36 (38.3)	3 (37.5)	0.964	34 (37.4)	5 (45.5)	0.602	26 (33.8)	13 (52.0)	0.103
**Interoceptive Deficits (EDI-3)**	19 (38.0)	35 (67.3)	0.003	33 (46.5)	21 (67.7)	0.048	48 (51.1)	6 (75.0)	0.193	47 (51.6)	7 (63.6)	0.452	38 (49.4)	16 (64.0)	0.202
**Emotional Dysregulation (EDI-3)**	18 (36.0)	31 (59.6)	0.017	27 (38.0)	22 (71.0)	0.002	44 (46.8)	5 (62.5)	0.394	42 (46.2)	7 (63.6)	0.273	37 (48.1)	12 (48.0)	0.996
**Perfectionism (EDI-3)**	15 (30.0)	25 (48.1)	0.062	28 (39.4)	12 (38.7)	0.945	35 (37.2)	5 (62.5)	0.160	33 (36.3)	7 (63.6)	0.079	26 (33.8)	14 (56.0)	0.048
**Asceticism (EDI-3)**	17 (34.0)	29 (55.8)	0.027	26 (36.6)	20 (64.5)	0.009	40 (42.6)	6 (75.0)	0.077	39 (42.9)	7 (63.6)	0.191	28 (36.4)	18 (72.0)	0.002
**Maturity Fears (EDI-3)**	19 (38.0)	33 (63.4)	0.010	34 (47.9)	18 (58.1)	0.344	48 (51.1)	4 (50.0)	0.954	45 (49.5)	7 (63.6)	0.374	32 (41.6)	20 (80.0)	0.001
**Total Problems (MASC 2)**	4 (8.0)	9 (17.3)	0.159	8 (11.3)	5 (16.1)	0.498	12 (12.8)	1 (12.5)	0.983	11 (12.1)	2 (18.2)	0.567	9 (11.7)	4 (16.0)	0.574
**Total score (CDI 2)**	16 (32.0)	30 (57.7)	0.009	27 (38.0)	19 (61.3)	0.030	42 (44.7)	4 (50.0)	0.772	40 (44.0)	6 (54.5)	0.505	31 (40.3)	15 (60.0)	0.085

The Pearson's chi-square test was used to evaluate the association between the presence of FA's symptoms (+ present; - absent) and clinical scores at YSR, EDI-3, MASC 2 and CDI 2 scales. Statistical significance for p<0-05. CDI 2, Children Depression Inventory 2; EDI-3, Eating Disorder Inventory- 3; MASC 2, Multidimensional Anxiety Scale for Children 2; YSR, Youth Self Report.
